# Myriocin enhances the antifungal activity of fluconazole by blocking the membrane localization of the efflux pump Cdr1

**DOI:** 10.3389/fphar.2022.1101553

**Published:** 2022-12-21

**Authors:** Hongkang Wang, Zhe Ji, Yanru Feng, Tianhua Yan, Yongbing Cao, Hui Lu, Yuanying Jiang

**Affiliations:** ^1^ Institute of Vascular Anomalies, Shanghai TCM-Integrated Hospital, Shanghai University of Traditional Chinese Medicine, Shanghai, China; ^2^ Department of Physiology and Pharmacology, School of Basic Medicine and Clinical Pharmacy, China Pharmaceutical University, Nanjing, China; ^3^ Department of Pharmacy, Shanghai Tenth People’s Hospital, School of Medicine, Tongji University, Shanghai, China

**Keywords:** efflux pump Cdr1, membrane localization, myriocin, fluconazole, *Candida albicans*

## Abstract

**Introduction:** Extrusion of azoles from the cell, mediated by an efflux pump Cdr1, is one of the most frequently used strategies for developing azole resistance in pathogenic fungi. The efflux pump Cdr1 is predominantly localized in lipid rafts within the plasma membrane, and its localization is sensitive to changes in the composition of lipid rafts. Our previous study found that the calcineurin signal pathway is important in transferring sphingolipids from the inner to the outer membrane.

**Methods:** We investigated multiple factors that enhance the antifungal activity of fluconazole (FLC) using minimum inhibitory concentration (MIC) assays and disk diffusion assays. We studied the mechanism of action of myriocin through qRT-PCR analysis and confocal microscopy analysis. We tested whether myriocin enhanced the antifungal activity of FLC and held therapeutic potential using a mouse infection model.

**Results:** We found that this signal pathway has no function in the activity of Cdr1. We found that inhibiting sphingolipid biosynthesis by myriocin remarkably increased the antifungal activity of FLC with a broad antifungal spectrum and held therapeutic potential. We further found that myriocin potently enhances the antifungal activity of FLC against *C. albicans* by blocking membrane localization of the Cdr1 rather than repressing the expression of Cdr1. In addition, we found that myriocin enhanced the antifungal activity of FLC and held therapeutic potential.

**Discussion:** Our study demonstrated that blocking the membrane location and inactivating Cdr1 by inhibiting sphingolipids biogenesis is beneficial for enhancing the antifungal activity of azoles against azole-resistant *C. albicans* due to Cdr1 activation.

## Introduction

Fluconazole (FLC), as a represent azole, is wildly used to treat invasive fungal infections due to its broad antifungal spectrum, good safety profile, and multiple administration routes (Lu et al., 2021). However, FLC is a fungistatic agent that cannot kill pathogenic fungi; fungi easily acquire azole resistance (Perlin et al., 2017). Extrusion of FLC from the cell, mediated by an efflux pump Cdr1, is one of the most frequently used strategies for developing FLC resistance in pathogenic fungi (Kim et al., 2019; Teo et al., 2019; Borgeat et al., 2021). Therefore, pharmacological inactivation of Cdr1 through suppressing the expression of Cdr1, blocking membrane localization of Cdr1, and inhibiting the combination of Cdr1 and antifungal agents, is beneficial to overcoming fungal FLC resistance (Monk and Goffeau, 2008). However, few compounds enhance the antifungal activity of FLC by inactivating Cdr1.

The activation of Cdr1 depends on the expression level of Cdr1, which is regulated by the Tac1 transcriptional regulator (Liu and Myers, 2017), and the membrane location of Cdr1, which is predominantly localized in lipid rafts within the plasma membrane (Pasrija et al., 2008). The activity and localization of Cdr1 are sensitive to changes in the composition of sphingolipids and ergosterol of lipid rafts (Pasrija et al., 2008). Our previous study showed that the calcineurin signaling pathway plays an important role in sphingolipid transport from the inner to the outer membrane. Still, it is unclear whether the influence on sphingolipid membrane transport can affect the integrity of lipid rafts and thus affect the membrane location and activity of Cdr1. Other factors that may affect the location and activity of the Cdr1 membrane also need further study.

In this study, we found that the calcineurin signaling pathway has no function in the activity of Cdr1. We further investigated multiple factors that enhance the antifungal activity of FLC. We found that inhibiting sphingolipid biosynthesis by myriocin remarkably increased the antifungal activity of FLC with a broad antifungal spectrum and held therapeutic potential. We further found that myriocin could enhance the antifungal activity of FLC by blocking membrane localization of Cdr1 rather than inhibiting the expression level of Cdr1. Our findings will help get specific small molecule inhibitors of Cdr1 and open the way for developing new antifungal therapeutics targeted at inhibiting the activity of Cdr1.

## Results

### Calcineurin has no function on the activation of Cdr1

**FIGURE 1:**
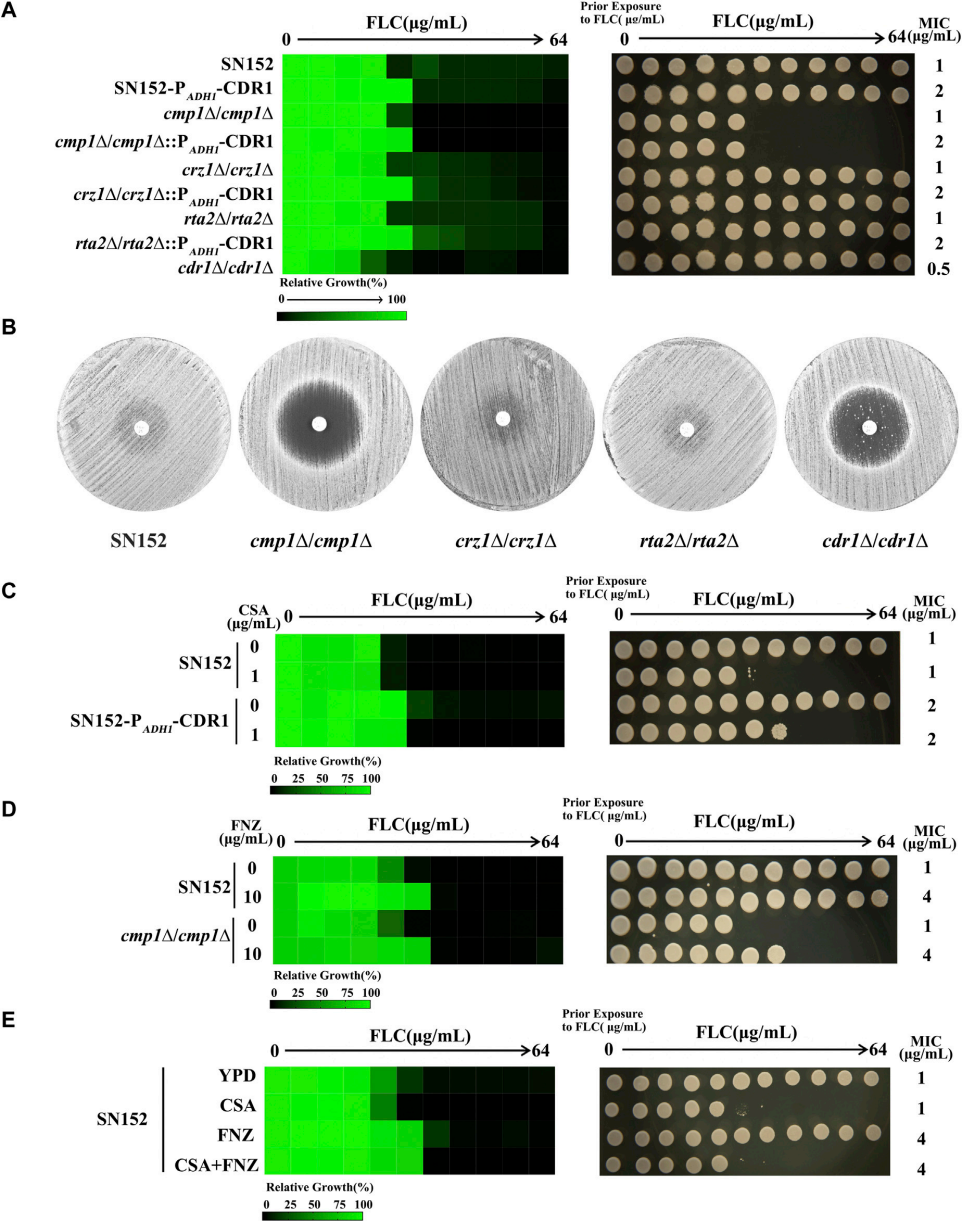
The impaired calcineurin signal pathway did not affect the function of Cdr1. **(A)** The sensitivities of *C. albicans* wild-type strain (SN152) and mutants (P_*ADH1*_-*CDR1*, *cmp1*∆/*cmp1*∆, *cmp1*∆/*cmp1*∆:: P_*ADH1*_-*CDR1*, *crz1*∆/*crz1*∆, *crz1*∆/*crz1*∆:: P_*ADH1*_-*CDR1*, *rta2*∆/*rta2*∆, *rta2*∆/*rta2*∆:: P_*ADH1*_-*CDR1*, *cdr1*∆/*cdr1*∆) to FLC were tested by the broth microdilution assays in a YPD medium incubated at 30°C for 48 h (Left). Cells from the broth microdilution assays were spotted onto YPD medium and incubated at 30°C for 48 h before the plate was photographed (Right). **(B)** Disk diffusion assays showed that the loss of the *CMP1* gene, but not the *CDR1* gene, cleared the inhibition zones of 25 μg FLC. In brief, cells (2 × 10^5^ cells) were spread onto YPD plates. A single 25 μg FLC disk (6 mm, Liofilchem, Italy) was placed in the center of each plate. Plates were then incubated at 30 C for 48 h before plates were photographed. **(C)** The MIC values of FLC of the SN152 strain and the P_*ADH1*_-*CDR1* mutant in a YPD medium without or with 1 μg/ml cyclosporin A were tested by the broth microdilution assays (at 30°C for 48 h) (Left). Cells from the broth microdilution assays were spotted onto YPD medium and incubated at 30°C for 48 h before the plate was photographed (Right). **(D)** The MIC values of FLC of the SN152 strain and the *cmp1*Δ/*cmp1*Δ null mutant in a YPD medium without or with 10 μg/ml fluphenazine (FNZ) were tested by the broth microdilution assays (at 30°C for 48 h) (Left). Cells from the broth microdilution assays were spotted onto YPD medium and incubated at 30°C for 48 h before the plate was photographed (Right). **(E)** The MIC values of FLC of the SN152 strain in a YPD medium with 1 μg/ml cyclosporin A, 10 μg/ml FNZ, 1 μg/ml cyclosporin A+10 μg/ml FNZ or without any compound (control) were tested by the broth microdilution assays (at 30°C for 48 h) (Left). Cells from the broth microdilution assays were spotted onto YPD medium and incubated at 30°C for 48 h before the plate was photographed (Right).

The Cdr1 is predominantly localized in lipid rafts, composed of sphingolipids and ergosterol (Hurst and Fratti, 2020). The calcineurin signal pathway transfers sphingolipids from the inner to the outer membrane (Jia et al., 2009). Therefore, we hypothesize that the calcineurin pathway may regulate the membrane localization and the activity of Cdr1 by affecting the transfer of sphingolipids and the composition of lipid rafts. To test this hypothesis, we successfully constructed null mutants of calcineurin signal pathway genes generating *cmp1*∆/*cmp1*∆, *crz1*∆/*crz1*∆, and *rta2*∆/*rta2*∆ null mutants (Supplementary Figure S1A). We also constructed the homogenesis gene deletion of the *CDR1* gene null mutant (*cdr1*∆/*cdr1*∆) (Supplementary Figure S1A). We found that loss of the Cdr1 led to increased susceptibility of *C. albica*ns to fluconazole (FLC). Compared to the wild-type strain, the minimum inhibitory concentration (MIC) value of FLC decreased from 1 to 0.5 μg/ml, but the *cdr1*∆/*cdr1*∆ null mutant could still grow in the presence of FLC (Figure 1A). However, contrary to the *cdr1*∆/*cdr1*∆, the *cmp1*∆/*cmp1*∆ mutant is inviable in the presence of FLC, rather than decreased FLC MIC value (Figure 1A). Losses of the Crz1 and Rta2 do not affect the susceptibility to FLC in *C. albicans* because that *crz1*∆/*crz1*∆ and *rta2*∆/*rta2*∆ null mutants and wild-type strain SN152 have the same value of FLC (1 μg/ml) and trailing growth in the presence of FLC (Figure 1A). Due to the tolerance of *C. albicans* to FLC, disk diffusion assays showed noticeable growth of cells in the zone of inhibition of 25 μg FLC on YPD plates incubated at 30°C for 48 h for SN152, *crz1*∆/*crz1*∆ null mutant, and *rta2*∆/*rta2*∆ null mutant (Figure 1B) (Rosenberg et al., 2018). It is worth noting that the zones of inhibition for 25 μg FLC treatments were clear on YPD plates for *cmp1*∆/*cmp1*∆ rather than for the *cdr*1∆/*cdr1*∆ null mutant (Figure 1B). The discrepant phenotypes between the *cdr1*Δ/*cdr1*Δ and *cmp1*∆/*cmp1*∆, *crz1*∆/*crz1*∆, and *rta2*∆/*rta2*∆ null mutants in susceptibility to FLC suggested that the calcineurin signal pathway has no function in the activation of Cdr1. To confirm this founding, we then created ectopic over-expression constructs of the *CDR1* gene in null mutants (*cmp1*∆/*cmp1*∆, *crz1*∆/*crz1*∆, and *rta2*∆/*rta2*∆) and wild-type strain (SN152) by expressing the *CDR1* gene using the potent *ADH1* promoter (Chang et al., 2018). We used PCR to verify the insertion position of the *ADH1* promoter in these mutants (Supplementary Figure S1B) and qRT-PCR to confirm the expression level of the *CDR1* gene (Supplementary Figure S1C). We found that over-expression of the *CDR1* gene can enhance the resistance of *C. albicans* to FLC in all null mutants and the wild-type strain because the MIC values of FLC increased from 1 to 2 μg/ml (Figure 1A). Further, we found that cyclosporin A, an inhibitor of a catalytic subunit of calcineurin (Sanglard et al., 2003), did not affect the increase of the MIC value of FLC caused by over-expression of the *CDR1* gene (Figure 1C) (Marchetti et al., 2003). Similarly, we induced over-expression of the *CDR1* gene by 10 μg/ml fluphenazine (FNZ) (Liu and Myers, 2017). We found that FNZ can increase the MIC value of FLC from 1 to 4 μg/ml against both the wild-type strain and the *cmp1*∆/*cmp1*∆ null mutant (Figure 1D), suggesting that the impaired calcineurin signal pathway cannot affect the function of Cdr1. Finally, in the presence of cyclosporin A (1 μg/mL), FNZ (10 μg/ml) can increase the MIC value of FLC against *C. albicans* (from 1 to 4 μg/ml) (Figure 1E); In the presence of FNZ (10 μg/ml), cyclosporine A (1 μg/ml) can still eliminate the FLC tolerance of *C. albicans* (Figure 1E). In summary, the calcineurin signal pathway functions in the transfer of sphingolipids from the inner to the outer membrane (Jia et al., 2009) and may affect the composition of lipid rafts but have no role in the activity of Cdr1.

### Myriocin enhanced the antifungal activity of FLC by inactivation of the Cdr1

The activation of Cdr1 plays an important role in the azole resistance in pathogenic fungi. Therefore, the inactivation of Cdr1 will enhance the antifungal activity of azoles against fungal infection. It is reported that ergosterol and sphingolipid contents can affect the membrane localization of Cdr1 (Pasrija et al., 2008). Therefore, we investigated the inactivation of FNZ-induced over-expression of the *CDR1* gene by sphingolipid biosynthesis inhibitors (myriocin and rapamycin) (He et al., 2004; Teixeira and Costa, 2016) and ergosterol biosynthesis inhibitors (cerulenin and terbinafine) (Nomura et al., 1972; Ryder, 1992). Previous studies demonstrated that geldanamycin [an inhibitor of heat shock protein 90 (Hsp90)] (Rosenberg et al., 2018), brefeldin A (a Golgi stress inducer) (Epp et al., 2010), tunicamycin (an endoplasmic reticulum stress inducer) (Sellam et al., 2009), staurosporine (a protein kinase C (PKC) inhibitor) (LaFayette et al., 2010), menadione (an oxidative stress inducer) (Sa et al., 2017), and tamoxifen (a calmodulin inhibitor) (Dolan et al., 2009), can enhance the antifungal activity of FLC. Still, it is unclear whether these compounds can affect the activity of Cdr1. In this study, we used 10 μg/ml FNZ to increase the MIC value of FLC from 1 to 4 μg/ml (Liu and Myers, 2017) and then examined which of these compounds could significantly reduce the MIC value of FLC against *C. albicans* in the presence of FNZ.

**FIGURE 2:**
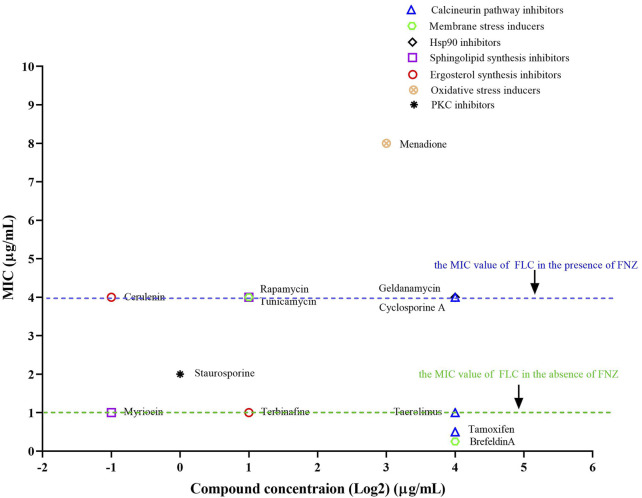
The synergistic antifungal activity of FLC and some compounds. Dose-matrix titration assays showed that some compounds (concentration range from 0.25 to 16 μg/ml) enhanced the antifungal activity of FLC in the presence of FNZ (10 μg/ml) (The MIC values of FLC reduced from 4 to 1 μg/ml or lower).

As discussed above, pharmacological compromise of the calcineurin pathway by cyclosporine A (16 μg/ml) did not change the MIC of FLC in the presence of FNZ (Figure 2, Supplementary Figure S2A). Tacrolimus binds to an FK506-binding protein (FKBP12) and inhibits calcineurin, and targets of rapamycin complex 1 (TORC1), which is important for ribosome biosynthesis (Azzi et al., 2013; Kasahara, 2021). Therefore, a high concentration of tacrolimus (16 μg/ml) inhibited both calcineurin and TORC1 and decreased the MIC value (from 4 to 1 μg/ml) of FLC in the presence of FNZ (Figure 2, Supplementary Figure S2B). Tamoxifen targets calmodulin (Dolan et al., 2009), then blocks the calcineurin pathway and inhibits NADPH-cytochrome P450 reductase Ccr1 (Liu et al., 2020) and ergosterol biosynthesis in fission yeast. Therefore, tamoxifen could improve the antifungal activity of FLC at a high concentration (16 μg/ml) (Figure 2, Supplementary Figure S2C) due to inhibiting ergosterol biosynthesis and then inactivation Cdr1 (Pasrija et al., 2008). Blocking the PKC pathway could enhance the antifungal activity of FLC and even make FLC fungicidal (LaFayette et al., 2010). We found that staurosporine (1 μg/ml) could decrease the MIC value of FLC from 4 to 2 μg/ml in the presence of FNZ (Figure 2, Supplementary Figure S2D), suggesting that the PKC pathway contributes to the activity of Cdr1. Hsp90 is an important molecular chaperone that regulates fungal drug resistance *via* physically interacts with the catalytic subunit of calcineurin and Mkc1 and maintains their stable conformations (Cowen, 2009; Singh et al., 2009; LaFayette et al., 2010). Indeed, geldanamycin made FLC act as fungicidal against *C. albicans* (Cowen and Lindquist, 2005; Rosenberg et al., 2018). However, in this study, we found that geldanamycin (16 μg/ml) did not decrease the MIC value of FLC in the presence of FNZ (Figure 2, Supplementary Figure S2E), suggesting that geldanamycin enhances the antifungal activity of FLC do not depend on inactivation of Cdr1. Membrane trafficking is important in maintaining cell membrane function and fungal drug resistance. Brefeldin A could inhibit the ADP ribosylation factor cycling, disrupt the membrane trafficking, and make FLC fungicidal against *C. albicans* (Epp et al., 2010). In the present study, we found that brefeldin A at a high concentration (16 μg/ml) could remarkably decrease the MIC value of FLC from 4 to 0.25 μg/ml (Figure 2 Supplementary Figure S2F), suggesting that the membrane trafficking process plays a vital role in the activity of Cdr1. It is reported that tunicamycin, as an endoplasmic reticulum stress inducer, had a synergistic antifungal activity with FLC against *C. albicans* (Sellam et al., 2009; Yu et al., 2013); however, which is not achieved through the inhibition of Cdr1 activity by tunicamycin (Figure 2 Supplementary Figure S2G). Oxidative stress could increase *C. albicans’* susceptivity to FLC (Xu et al., 2009). However, in the presence of FNZ, menadione (8 μg/ml), as an oxidative stress inducer, antagonizes the antifungal activity of FLC (the MIC value of FLC increased from 4 to 8 μg/ml) (Figure 2, Supplementary Figure S2H). Intracellular ergosterol is important for membrane localization and the activity of Cdr1 (Pasrija et al., 2008). We found that terbinafine (an inhibitor of Erg1) (Ryder, 1992) could decrease the MIC value of FLC in the presence of FNZ (Figure 2, Supplementary Figure S2I), but cerulenin (an inhibitor of 3-hydroxy-3-methylglutaryl coenzyme A) (Nomura et al., 1972) could not (Figure 2, Supplementary Figure S2J). In addition, sphingolipids also play an important role in *C. albicans*’ resistance to FLC (Gao et al., 2018). However, rapamycin, as an inhibitor of the target of rapamycin (TOR) signal pathway and able to inhibit sphingolipid synthesis (Teixeira and Costa, 2016), did not enhance the antifungal activity of FLC in the presence of FNZ (Figure 2, Supplementary Figure S2K). Notably, myriocin, which is an inhibitor of serine-palmitoyl-transferase that is essential for sphingolipid synthesis (He et al., 2004), at a concentration as low as 0.5 μg/ml could significantly reduce the MIC value of FLC from 4 to 1 μg/ml in the presence of FNZ (Figure 2, Supplementary Figure S2L). To sum up, myriocin is the most potential adjuvant to enhance the antifungal activity of FLC.

**FIGURE 3:**
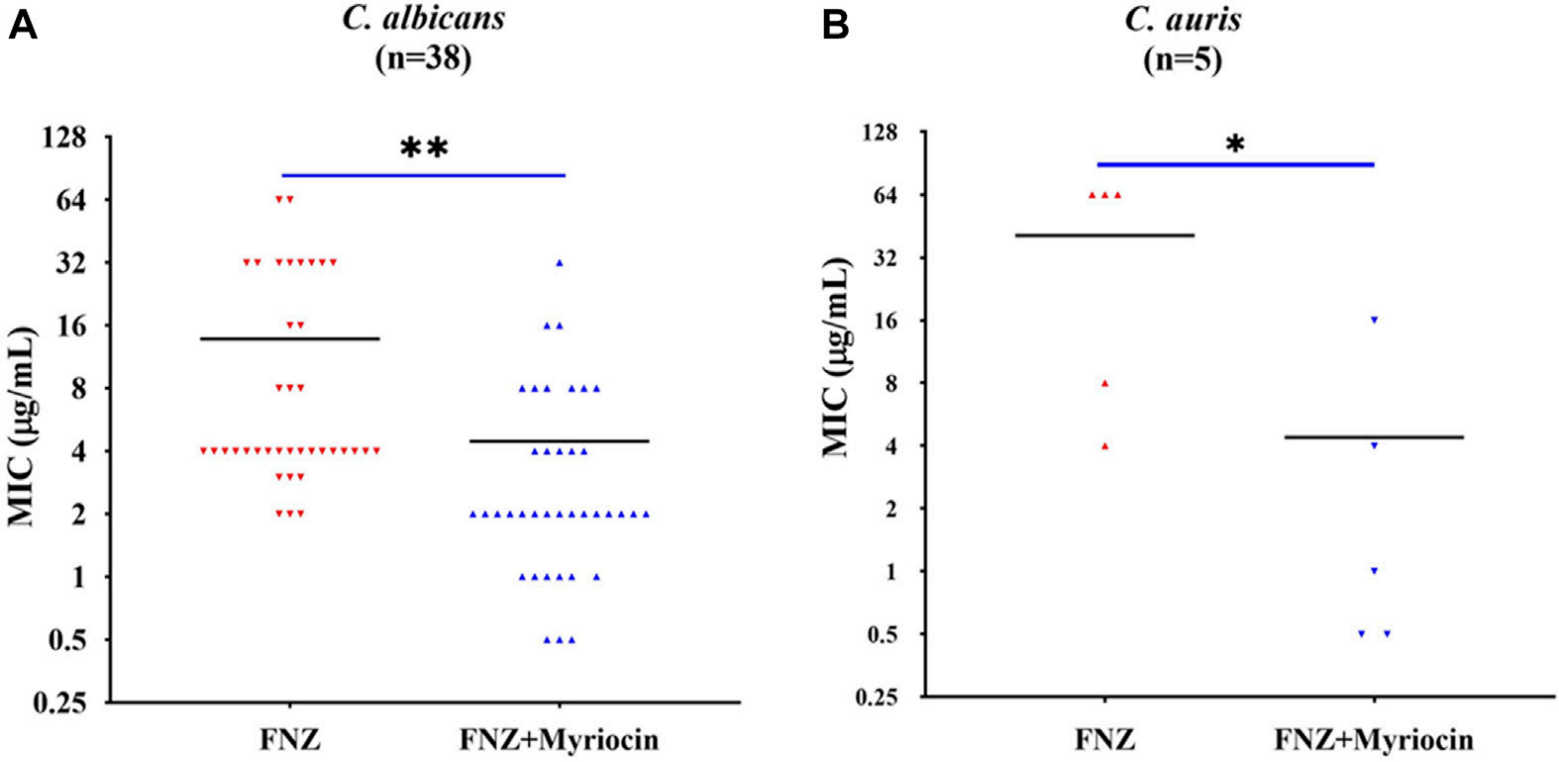
Myriocin can significantly reduce theMIC values of FLC against **(A)** *C. albicans* isolates (*n* = 38) (**p = 0.0017, the FNZ (10 μg/ml) + myriocin (0.5 μg/ml) treated group compared to the FNZ (10 μg/ml) group, t-Test) and **(B)** *C. auris* isolates (*n* = 5) (*p = 0.036, the FNZ (10 μg/ml) + myriocin (0.5 μg/ml) treated group compared to the FNZ (10 μg/ml) group, t-Test).

To examine whether the inhibitory effect of myriocin on the activity of the Cdr1 enhancing the antifungal activity of FLC is conserved across other *C. albicans* strains and pathogenic *Candida* species, we tested the antifungal activity of FLC plus 0.5 μg/ml myriocin combinations compared to FLC alone in the presence of 10 μg/ml FNZ in clinical isolates of *C. albicans* (*n* = 38), *C. auris* (*n* = 5) *C. glabrata* (*n* = 13), *C. krusei* (*n* = 9), *C. parapsilosis* (*n* = 43), and *C. tropicalis* (*n* = 12). Myriocin significantly reduced the MIC value of FLC against *C. albicans* and *C. auris* strains (Figure 3). However, myriocin did not enhance the antifungal activity of FLC against *C. glabrata*, *C. krusei*, *C. parapsilosis*, and *C. tropicalis* (Supplementary Figure S3).

### Myriocin inactivated the Cdr1 *via* blocking membrane localization of Cdr1

**FIGURE 4:**
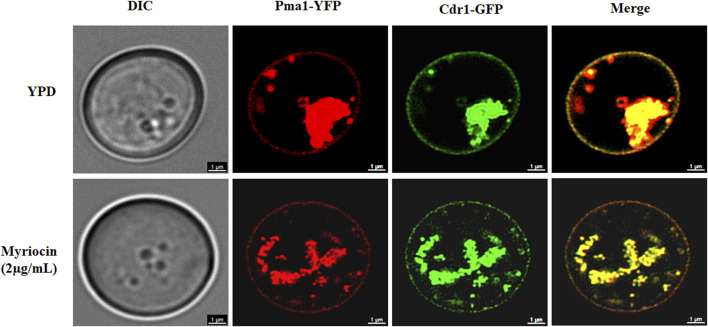
Confocal micrographs of cell membrane staining (Pma1-YFP) and membrane localization of Cdr1-GFP in the Cdr1-GFP:: Pma1-YFP mutant after treatment with or without 2 μg/mL myriocin for 16 h in YPD medium. DIC, differential interference contrast; YFP, yellow fluorescent protein; GFP, green fluorescent protein. Scale bar = 1 μm.

We speculated that myriocin might inactivate Cdr1 by inhibiting the expression or membrane localization of Cdr1. We tested the expression level of the *CDR1* gene by quantitative real-time PCR (qRT-PCR) analysis. Compared with FNZ treated (10 μg/ml) or untreated *C. albicans* cells, myriocin (0.5 μg/ml) treated *C. albicans* cells had significantly higher expression of the *CDR1* gene (Supplementary Figure S4). The qRT-PCR analysis demonstrated that myriocin induces the expression of the *CDR1* gene rather than suppresses its expression, suggesting that myriocin inactivated Cdr1 by blocking membrane localization of Cdr1 and consequently compensatively causing the expression of the *CDR1* gene. To verify this hypothesis, we tagged the C-termini of Cdr1 with a GFP tag (Chang et al., 2018) (Supplementary Figure S5A) and Pma1 (a marker protein of lipid rafts) (Shukla et al., 2003) with a YFP tag (Gola et al., 2003) (Supplementary Figure S5B). Without myriocin, like Pma1, Cdr1 is located on the cell membrane. However, in the presence of myriocin (2 μg/ml), the amount of Cdr1 located on the membrane is reduced (Figure 4). In summary, these results suggested that myriocin inactivated Cdr1 and enhanced the antifungal activity of FLC by blocking membrane localization of Cdr1 rather than suppressing the expression of Cdr1.

### Myriocin enhanced the antifungal activity of FLC against invasive infection caused by *C. albicans*

**FIGURE 5:**
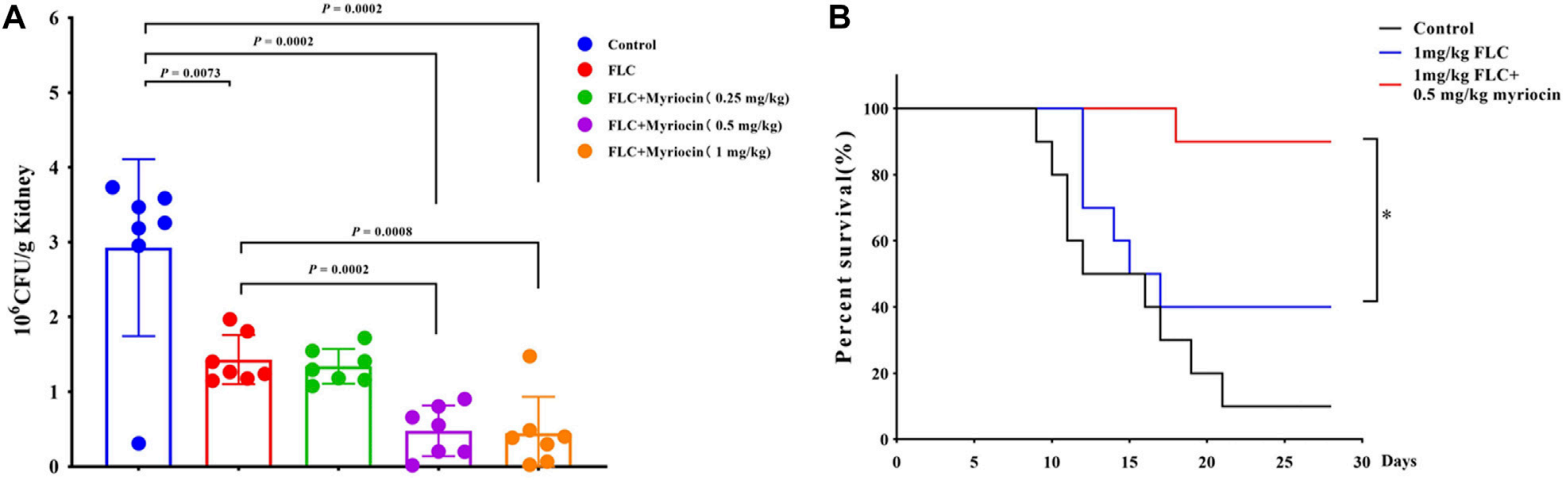
Myriocin enhances the antifungal activity against *C. albicans in vivo*. **(A)** Kidney CFU assay in mice with systemic candidiasis after treatment with PBS, FLC (1 mg/kg), FLC (1 mg/kg) + myriocin (0.25 mg/kg), FLC (1 mg/kg) + myriocin (0.5 mg/kg), and FLC (1 mg/kg) + myriocin (1 mg/kg) for 3 days. **(B)** Survival curves of C57BL/6 mice infected by SN152 and treated with FLC (1 mg/kg) or FLC (1 mg/kg) + myriocin (0.25 mg/kg) for 3 days, *p = 0.0137 (Log-rank test).

We employed a mouse candidiasis model to evaluate whether myriocin enhances the antifungal activity against *C. albicans in vivo*. Female C57BL/6 mice have been infected with *C. albicans* (the wild-type SN152 strain) cells *via* tail vein injection. After 2 h of infection, PBS, FLC (1 mg/kg), FLC (1 mg/kg) + myriocin (0.25 mg/kg), FLC (1 mg/kg) + myriocin (0.5 mg/kg), and FLC (1 mg/kg) + myriocin (1 mg/kg) treatment was given intraperitoneally. The antifungal drug treatment lasted for 3 days. After being infected for 5 days, seven mice from each group were euthanized and enumerated for *C. albicans’* burden in kidneys. Lower kidney fungal burden was observed from the kidneys of mice treated with FLC compared to a control group (*p* = 0.0073, *t*-test) (Figure 5A). Of note, the fungal burden was significantly lower after being treated with the FLC (1 mg/kg) + myriocin (0.5 mg/kg) treated group (*p* = 0.0002, *t*-test) and FLC (1 mg/kg) + myriocin (1 mg/kg) treated group (*p* = 0.0008, *t*-test) in comparison with the FLC (1 mg/kg) treated group (Figure 5A).

**FIGURE 6:**
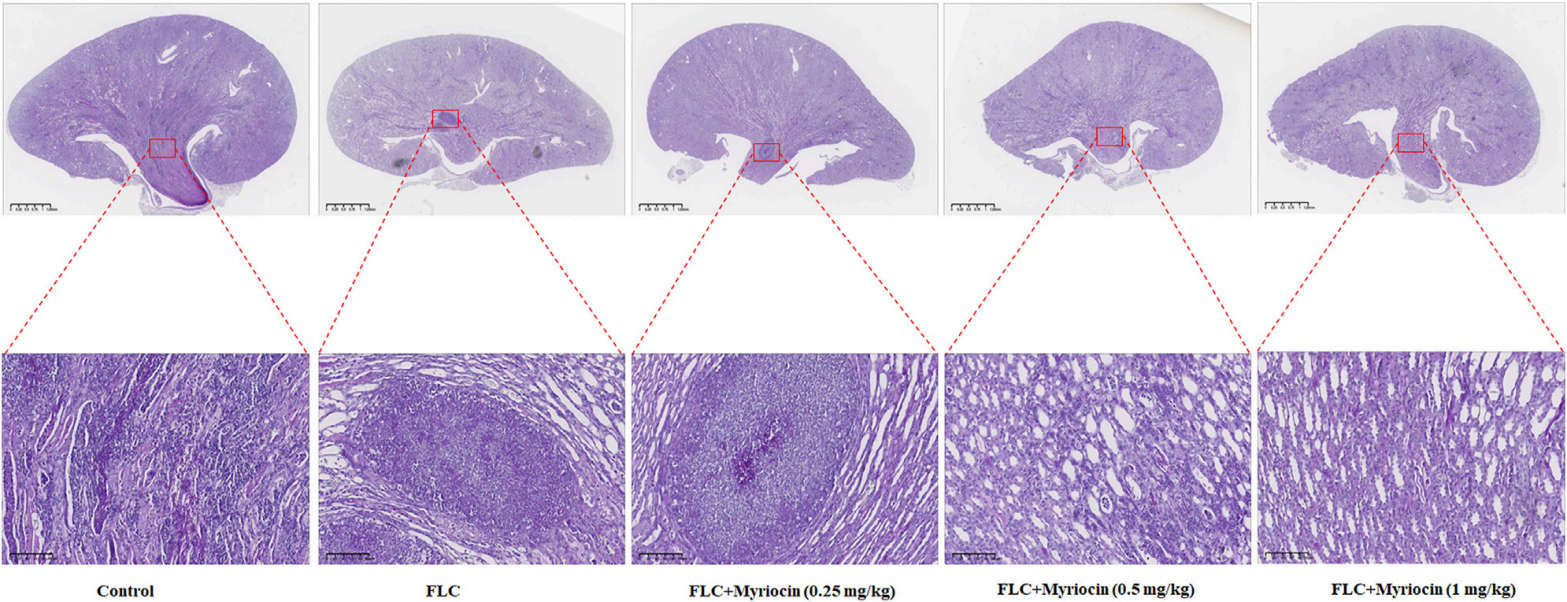
Kidneys from mice (*n* = 6 per group) were fixed and stained with periodic acid Schiff stain for histopathology. Scale bar, 100 μm/1.25 mm. The experiment was performed in biological triplicate.

To test whether myriocin enhanced the antifungal activity of FLC and held therapeutic potential, we randomly divided the mice into three groups 1) no drug-treated group (as control), 2) 1 mg/kg FLC treated group, and 3) 1 mg/kg FLC plus 0.5 mg/kg myriocin treated group, with ten mice in each group. We found that the mortality of the control group was 90% during the 28-day observation, and the median survival time of the control group was 14 days (Figure 5B). After being treated with 1 mg/kg FLC, the mortality of infected mice decreased to 60%, and the median survival time extended to 16 days (*p*-value is 0.1533 for comparison between FLC treated group and control group. Log-rank test) (Figure 5B). It is worth noting that myriocin (0.5 mg/kg) enhanced the antifungal activity of FLC (1 mg/kg) against *C. albicans* infection because the mortality of 1 mg/kg FLC plus 0.5 mg/kg myriocin-treated group decreased to 10% (Figure 5B) (*p*-value is 0.0002 for comparison between FLC plus myriocin treated group and control group. *p*-value is 0.0137 for comparison between FLC plus myriocin-treated and FLC-treated groups. Log-rank test). For histopathology, the kidneys of mice were fixed and stained with periodic acid Schiff (PAS) stain. Compared to no drug-treated group, after FLC (1 mg/kg) or FLC (1 mg/kg) + myriocin (0.25 mg/kg) treatment, the number of fungal infection lesions in the kidneys of mice infected with *C. albicans* decreased, but remained. In contrast, after treatment with FLC (1 mg/kg) and myriocin (0.5 mg/kg or 1 mg/kg) combination, the infection focuses of the fungal-infected mouse kidney disappeared (Figure 6). In summary, these *in vivo* experiments suggested that myriocin enhanced the antifungal activity of FLC against fungal infection caused by *C. albicans*.

## Discussion

FLC is widely used to treat invasive fungal infections because of its broad antifungal spectrum, well safety profile, and multiple routes of administration. However, it is easy for fungi to obtain FLC resistance because FLC is a fungistatic agent (Lu et al., 2021). The activation of Cdr1 and the reduction of intracellular FLC concentration is one of the important mechanisms of FLC resistance in fungi (Monk and Goffeau, 2008). There are three strategies to inactivate Cdr1: 1) inhibiting the expression of Cdr1, 2) using azole analogs to inhibit the binding of azoles to Cdr1, and 3) blocking membrane localization of Cdr1. Indeed, loss of Cdr1 resulted in remarkably increased susceptibility of *C. albicans* to FLC, miconazole, ketoconazole, and itraconazole (Sanglard et al., 1996; Jha et al., 2004; Xu et al., 2007; Tsao et al., 2009; Xu et al., 2021). Similarly, the efflux pump protein Cdr1 also plays an important role in the azole resistance of *C. glabrata* (Galkina et al., 2020), *C. auris* (Carolus et al., 2021), *Candida lusitaniae* (Borgeat et al., 2021), *Fusarium keratoplasticum* (James et al., 2021). In this study, myriocin significantly enhanced the antifungal activity of FLC by blocking membrane localization and inactivating Cdr1. Therefore, the inactivation of Cdr1 is an important and promising antifungal strategy (Prasad et al., 2019).

Sphingolipids play an important role in fungal azole resistance (Song et al., 2020). Altering sphingolipid composition makes *C. albicans* gain azole resistance (Gao et al., 2018). Our previous study found that blocking the transfer of sphingolipids from the inner to the outer membrane by genetic inactivation (deletion of the *RTA2* gene) increased the FLC susceptibility of *C. albicans* (Jia et al., 2009). Inhibiting sphingolipid and ergosterol biosynthesis can change the composition of lipid rafts in the plasma membrane and block the membrane localization of Cdr1 (Pasrija et al., 2005; Prasad et al., 2005; Pasrija et al., 2008). In *C. albicans*, the deletion of the *ERG11* gene led to ergosterol deficiency and a decrease in plasma membrane fluidity (Suchodolski et al., 2019). In the *erg11* ∆/*erg11*∆ null mutant, Cdr1 falls off the plasma membrane to the vacuole in the early logarithmic growth phase, and there is a positioning error (Suchodolski et al., 2019). Similarly, lactic acid can reduce the expression of the *ERG11* gene, thereby affecting the location of Cdr1 and blocking the activity of Cdr1 (Suchodolski et al., 2021). In *C. albicans*, when functional mitochondria are damaged, Cdr1 will be misplaced on the vacuole membrane (Thomas et al., 2013), indicating that functional mitochondria exert post-translational regulation on the level of Cdr1, thus affecting the biological function of Cdr1. The research on the role of Cdr1 in *C. albicans* shows that when cysteine at positions 1056, 1091, 1106 and 1294 is replaced separately, Cdr1 cannot be correctly located on the cell membrane (Prasad et al., 2012). In this study, we found that the calcineurin signaling pathway does not affect the activity of Cdr1, suggesting that the process of sphingolipid transfer has little effect on the integrity of lipid rafts and the membrane localization of Cdr1. In this study, we used FNZ to induce the expression of Cdr1 and increase the MIC values of FLC against pathogenic fungi (Liu and Myers, 2017). We found that pharmacological compromise of sphingolipid biosynthesis by myriocin could occur in the inactivation of Cdr1. It is worth noting that myriocin at sub-MIC could significantly enhance the antifungal activity against clinical *C. albicans* isolates.

Our present study demonstrated that inhibiting sphingolipid biosynthesis by myriocin remarkably increased the antifungal activity of FLC with a broad antifungal spectrum and held therapeutic potential. The synergistic antifungal activity of FLC and myriocin depends on the fact that myriocin blocks membrane localization of Cdr1. Our findings will help overcome the fungal azole resistance caused by Cdr1 activation and open the way for developing new antifungal therapeutics targeted at inhibiting the activity of Cdr1.

## Materials and methods

### Strains, primers, agents, and cultural conditions

All strains used in this study are listed in Supplementary Table S1. All primers used in this study are listed in Supplementary Table S2. We routinely used a YPD medium (1% (W/V) yeast extract, 2% (W/V) peptone, and 2% (W/V) dextrose) to culture *Candida* strains at 30 °C. To prepare the solid medium plates, we added 2% (W/V) agar to the liquid medium. To construct mutant strains, we used a synthetic complete dropout medium (0.67% (W/V) yeast nitrogen base without amino acids, 2% (W/V) dextrose, 2% (W/V) agar, and appropriate amino acid mix) to screen positive colonies. Drug stock solutions were prepared using dimethyl sulfoxide (DMSO) (Sangon Biotech, Shanghai, China) as a solvent for brefeldin A (6.4 mg/ml) (MCE, Shanghai, China), cerulenin (6.4 mg/ml) (MCE, Shanghai, China), cyclosporin A (6.4 mg/ml) (Aladdin, Shanghai, China), FLC (6.4 mg/ml) (Aladdin, Shanghai, China), FNZ (6.4 mg/ml) (MCE, Shanghai, China), geldanamycin (Sangon Biotech, Shanghai, China), menadione (Aladdin, Shanghai, China), myriocin (6.4 mg/ml) (MCE, Shanghai, China), rapamycin (6.4 mg/ml) (MCE, Shanghai, China), staurosporine (6.4 mg/ml) (MCE, Shanghai, China), tacrolimus (6.4 mg/ml) (Aladdin, Shanghai, China), and terbinafine (6.4 mg/ml) (MCE, Shanghai, China).

### MIC assays

MIC assays were carried out according to a modified version of the CLSI procedure (M27, fourth edition). Briefly, 100 μl of drugs at two-fold the final concentrations were serially diluted in YPD medium in 96-well culture plates and combined with 100 μl of fungal cultures in which the fungal cell concentration was adjusted to 1×10^3^ cells/ml. Plates were incubated at 30°C, and optical densities were read after 48 h with a Spectra Max ID3 plate reader (Molecular Devices, MD, United States); the MIC is defined as the first well with more than 50% growth reduction in terms of OD_600_ values compared to the drug-free cells. Before the 48 h OD_600_ readings, we carefully shook the plates and spotted a representative aliquot of 5 μl of each well on fresh YPD solid medium plates. Recovery plates were incubated at 30°C for 48 h before being photographed. All assays were performed and repeated three times.

### Disk diffusion assays

We carried out disk diffusion assays according to the CLSI M44-A2 guidelines with slight modifications. In short, strains were cultured overnight in a YPD medium at 30°C, cell density was adjusted to 1 × 10^6^ cells/ml in sterile PBS, and 100 μl of cell suspension was streaked on YPD solid medium plates. One paper disk supplemented with 25 μg FLC (Liofilchem, Italy) was placed in the center of each plate. The plates were then incubated for 48 h and photographed.

### RNA extractions and quantitative real-time PCR assays

*C. albicans* strains were grown overnight in YPD medium at 30°C with shaking. Total RNA was extracted using a YeaStar RNA Kit (ZymoResearch, United States). Reverse transcription of the isolated RNA samples was performed by using the PrimeScript™ RT reagent Kit with gDNA Eraser (Takara Bio, Japan). The cDNA abundance was relatively quantified using TB Green^®^ Premix DimerEraser™ (Takara Bio, Japan) in a CFX96™ Real-Time PCR Detection System (Bio-Rad, United States) with the following strategy: 1) 95°C for 30 s; 2) 95°C for 5 s, 50°C for 30 s, and 72°C for 30 s, for 40 cycles. The relative expression level of the *CDR1* gene was normalized to that of the reference *ACT1* gene, and the data were interpreted as fold changes based on the untreated control according to the 2^−ΔΔCt^ method and triplicate measurements were conducted with each sample (Lu et al., 2015).

### Disruption of target genes

We used a fusion PCR method to delete the two alleles of target genes from the strain SN152 (Noble and Johnson, 2005). Briefly, the first round of PCR reactions involved the amplification of the flanking sequences (approximately 350-bp) of target genes (with a genomic DNA of SN152 strain and primers P1 and P3 or P4 and P6, in separate reactions) and the selectable marker (*HIS1* or *ARG4*) with a template of plasmid pSN52 or pSN69 and primers universal primer two and universal primer 5. We used primers P1 and P6 to amplify gene deletion cassettes with all three first-round PCR products. We then transformed gene deletion cassettes into the SN152 strain or heterozygous mutant strain using Yeastmaker™ Yeast Transformation System two kit (Clontech Laboratories, United States) and selected on synthetic media containing the necessary auxotrophic supplements for heterozygous or homozygous mutant strains. The primers used for diagnosis of target genes knockouts were, for the 5′ junctions, a primer UCheck plus a primer HIS1left or ARG4left; for the 3′ junctions, a primer Dcheck plus a primer HIS1right or ARG4right.

### Over-expression of the *CDR1* gene

The over-expressed *CDR1* gene mutant was constructed (Chang et al., 2018). The pCPC158 backbone is amplified using F1/R1 primers, generating a product with a 39 bp flank homology region to the *CDR1* gene. Using this PCR product as a PCR template, the second round of PCR using F2/R2 primers generates the ectopic expression cassette, an N-terminal tagging cassette with 78 bp homology regions to the *CDR1* gene. After transformation and integration, the constitutive *ADH1* promoter located upstream of the *CDR1* gene increased the expression of the *CDR1* gene. Verification primers VP42 and VP43 were used for diagnostic PCR.

### C-terminal of proteins tagging GFP and YFP

To tag the C-terminal of Cdr1 using GFP, we adopted a PCR strategy to amplify the desired DNA cassettes in a plasmid pCPC64 (Chang et al., 2018). For the first round of PCR using F1/R1 primers, a product with 39 bp homology regions is generated. Using this product as a PCR template directly, the second round of PCR using F2/R2 primers yields DNA cassettes with 78 bp homology regions to the *CDR1* gene. This product could be transformed into *C. albicans* cells to generate a mutant with the C-terminal tagged Cdr1 with GFP. We used primers Cdr1CUpCheck plus VP8 to check the 5′ integration and VP19 plus Cdr1CDnCheck for the 3′ integration. We further tagged the C-terminal of Pma1 with YFP in the Cdr1-GFP mutant using a pFA-YFP-ARG4 plasmid as described previously (Gola et al., 2003). Diagnostic PCR used primers Pma1Upcheck plus A2 and A3 plus Pma1Dncheck to confirm the C-terminal of Pma1 tagged with YFP mutant.

### Confocal microscopy analysis

The effect of myriocin on the membrane localization of Cdr1 was determined by a confocal laser scanning microscopy (Stellarissted, Leica, Germany) in the Cdr1-GFP::Pma1-YFP mutant. *C. albicans* cells were cultured at 30°C and treated without or with 2 μg/ml myriocin for 16 h. The fluorescence of GFP was excited by the laser of 488 nm with an emission of 500–560 nm, and the fluorescence of YFP was excited by the laser of 510 nm with an emission of 527 nm.

### Mouse infection model

Groups of C57BL/6 female mice (6–8 weeks) were inoculated *via* lateral tail vein with 100 μl PBS containing 1 × 10^5^ *C. albicans* cells. FLC (1 mg/kg) and myriocin (0.5 mg/kg) were administered to the infected mice once a day intraperitoneally for 3 days, starting 2 h after the injection with *C. albicans*. Mice were monitored daily for survival for 28 days. Kaplan-Meier –analyses were used to indicate the survival probabilities, and Log-rank testing was used to evaluate the significance of survival curves. The mice were sacrificed 2 days after the last administration; the left kidney of each mouse was taken and homogenized in sterile PBS, diluted and coated on the YPD solid medium plates, and incubated at 30°C for 48 h before counting colonies; The right kidney of the animal was taken for histopathology by periodic acid-Schiff (PAS) staining to visualize the fungal burden. The Tongji University Animal Care Committee approved all experimental procedures involving animals (No.: TJAA00322102).

## Data Availability

The raw data supporting the conclusion of this article will be made available by the authors, without undue reservation.

## Supplementary material

SUPPLEMENTARY FIGURE S1 Deletions of target genes (the CMP1, CRZ1, RTA2, CDR1 genes) were diagnosed by PCR, for the 5′ junctions, a primer UCheck plus a primer HIS1left or ARG4left; **(A)** for the 3′ junctions, a primer Dcheck plus a primer HIS1right or ARG4right. **(B)** PADH1-CDR1, cmp1Δ/cmp1Δ:: PADH1-CDR1, crz1Δ/crz1Δ: PADH1-CDR1, and rta2Δ/rta2Δ:: PADH1-CDR1 mutants ectopic over-expression the CDR1 gene were checked by the primers Cdr1NUpcheck plus VP42 and the primers VP43 plus Cdr1NDncheck. **(C)** We determined the expression of the CDR1 gene in the wild-type strain (SN152) and mutants using qRT-PCR.

SUPPLEMENTARY FIGURE S2 Dose-matrix titration assays (incubated in a YPD medium at 30℃ for 48 h) were used to evaluate the synergistic antifungal activity of FLC and antifungal agents, **(A)** including cyclosporine A, **(B)** tacrolimus, **(C)** Tamoxifen, **(D)** staurosporine, **(E)** geldanamycin, **(F)** brefeldin A, **(G)** tunicamycin, **(H)** menadione, **(I)** terbinafine, **(J)** cerulenin, **(K)** rapamycin, **(L)** myriocin.

SUPPLEMENTARY FIGURE S3 Myriocin did not enhance the antifungal activity of FLC against *C. glabrata* (*n* = 13), *C. krusei* (*n* = 9), *C. parapsilosis* (*n* = 43), and *C. tropicalis* (*n* = 12).

SUPPLEMENTARY FIGURE S4 The qRT-PCR analysis demonstrated that myriocin (0.5 μg/mL) induced the expression of the *CDR1* gene.

SUPPLEMENTARY FIGURE S5
**(A)** The C-termini of Cdr1 with a GFP tag mutant was checked by PCR using the Cdr1CUpCheck plus VP8 and the primers VP19 plus Cdr1CDnCheck. **(B)** The C-termini of Pma1 with a YFP tag mutant was diagnosed by PCR using the primers Pma1UpCheck plus A2 and the primers A3 plus Pma1DnCheck.

## References

[B1] Azzi J. R. Sayegh M. H. Mallat S. G. (2013). Calcineurin inhibitors: 40 years later, can't live without. J. Immunol. 191, 5785–5791. 10.4049/jimmunol.1390055 24319282

[B2] Borgeat V. Brandalise D. Grenouillet F. Sanglard D. (2021). Participation of the ABC transporter CDR1 in azole resistance of Candida lusitaniae. J. Fungi (Basel) 7, 760. 10.3390/jof7090760 34575798 PMC8467326

[B3] Carolus H. Pierson S. Munoz J. F. Subotic A. Cruz R. B. Cuomo C. A. (2021). Genome-Wide analysis of experimentally evolved Candida auris reveals multiple novel mechanisms of multidrug resistance. mBio 12, 033333–e3420. 10.1128/mBio.03333-20 PMC8092288 33820824

[B4] Chang P. Wang W. Igarashi Y. Luo F. Chen J. (2018). Efficient vector systems for economical and rapid epitope-tagging and overexpression in Candida albicans. J. Microbiol. Methods 149, 14–19. 10.1016/j.mimet.2018.04.016 29698691

[B5] Cowen L. E. (2009). Hsp90 orchestrates stress response signaling governing fungal drug resistance. PLoS Pathog. 5, e1000471. 10.1371/journal.ppat.1000471 19714223 PMC2726949

[B6] Cowen L. E. Lindquist S. (2005). Hsp90 potentiates the rapid evolution of new traits: Drug resistance in diverse fungi. Science 309, 2185–2189. 10.1126/science.1118370 16195452

[B7] Dolan K. Montgomery S. Buchheit B. Didone L. Wellington M. Krysan D. J. (2009). Antifungal activity of tamoxifen: *In vitro* and *in vivo* activities and mechanistic characterization. Antimicrob. Agents Chemother. 53, 3337–3346. 10.1128/AAC.01564-08 19487443 PMC2715577

[B8] Epp E. Vanier G. Harcus D. Lee A. Y. Jansen G. Hallett M. (2010). Reverse genetics in Candida albicans predicts ARF cycling is essential for drug resistance and virulence. PLoS Pathog. 6, e1000753. 10.1371/journal.ppat.1000753 20140196 PMC2816695

[B9] Galkina K. V. Okamoto M. Chibana H. Knorre D. A. Kajiwara S. (2020). Deletion of CDR1 reveals redox regulation of pleiotropic drug resistance in Candida glabrata. Biochimie 170, 49–56. 10.1016/j.biochi.2019.12.002 31843579

[B10] Gao J. Wang H. Li Z. Wong A. H. Wang Y. Z. Guo Y. (2018). Candida albicans gains azole resistance by altering sphingolipid composition. Nat. Commun. 9, 4495. 10.1038/s41467-018-06944-1 30374049 PMC6206040

[B11] Gola S. Martin R. Walther A. Dunkler A. Wendland J. (2003). New modules for PCR-based gene targeting in Candida albicans: Rapid and efficient gene targeting using 100 bp of flanking homology region. Yeast 20, 1339–1347. 10.1002/yea.1044 14663826

[B12] He Q. Johnson V. J. Osuchowski M. F. Sharma R. P. (2004). Inhibition of serine palmitoyltransferase by myriocin, a natural mycotoxin, causes induction of c-myc in mouse liver. Mycopathologia 157, 339–347. 10.1023/b:myco.0000024182.04140.95 15180163

[B13] Hurst L. R. Fratti R. A. (2020). Lipid rafts, sphingolipids, and ergosterol in yeast vacuole fusion and maturation. Front. Cell Dev. Biol. 8, 539. 10.3389/fcell.2020.00539 32719794 PMC7349313

[B14] James J. E. Lamping E. Santhanam J. Cannon R. D. Abd Razak M. F. Zakaria L. (2021). A 23 bp cyp51A promoter deletion associated with voriconazole resistance in clinical and environmental isolates of neocosmospora keratoplastica. Front. Microbiol. 12, 272. 10.3389/fmicb.2020.00272 PMC7136401 32296397

[B15] Jha S. Dabas N. Karnani N. Saini P. Prasad R. (2004). ABC multidrug transporter Cdr1p of Candida albicans has divergent nucleotide-binding domains which display functional asymmetry. FEMS Yeast Res. 5, 63–72. 10.1016/j.femsyr.2004.07.002 15381123

[B16] Jia X. M. Wang Y. Jia Y. Gao P. H. Xu Y. G. Wang L. (2009). RTA2 is involved in calcineurin-mediated azole resistance and sphingoid long-chain base release in Candida albicans. Cell Mol. Life Sci. 66, 122–134. 10.1007/s00018-008-8409-3 19002381 PMC11131505

[B17] Kasahara K. (2021). Physiological function of FKBP12, a primary target of rapamycin/FK506: A newly identified role in transcription of ribosomal protein genes in yeast. Curr. Genet. 67, 383–388. 10.1007/s00294-020-01142-3 33438053

[B18] Kim S. H. Iyer K. R. Pardeshi L. Munoz J. F. Robbins N. Cuomo C. A. (2019). Genetic analysis of Candida auris implicates Hsp90 in morphogenesis and azole tolerance and Cdr1 in azole resistance. mBio 10, 025299–e2618. 10.1128/mBio.02529-18 PMC6355988 30696744

[B19] LaFayette S. L. Collins C. Zaas A. K. Schell W. A. Betancourt-Quiroz M. Gunatilaka A. A. (2010). PKC signaling regulates drug resistance of the fungal pathogen Candida albicans via circuitry comprised of Mkc1, calcineurin, and Hsp90. PLoS Pathog. 6, e1001069. 10.1371/journal.ppat.1001069 20865172 PMC2928802

[B20] Liu Q. Guo X. Jiang G. Wu G. Miao H. Liu K. (2020). NADPH-cytochrome P450 reductase Ccr1 is a target of tamoxifen and participates in its antifungal activity via regulating cell wall integrity in fission yeast. Antimicrob. Agents Chemother. 64, 000799–e120. 10.1128/AAC.00079-20 PMC7449163 32571823

[B21] Liu Z. Myers L. C. (2017). Mediator tail module is required for tac1-activated CDR1 expression and azole resistance in Candida albicans. Antimicrob. Agents Chemother. 61, 013422–e1417. 10.1128/AAC.01342-17 PMC5655045 28807920

[B22] Lu H. Shrivastava M. Whiteway M. Jiang Y. (2021). Candida albicans targets that potentially synergize with fluconazole. Crit. Rev. Microbiol. 47, 323–337. 10.1080/1040841x.2021.1884641 33587857

[B23] Lu H. Yao X. W. Whiteway M. Xiong J. Liao Z. B. Jiang Y. Y. (2015). Loss of RPS41 but not its paralog RPS42 results in altered growth, filamentation and transcriptome changes in Candida albicans. Fungal Genet. Biol. 80, 31–42. 10.1016/j.fgb.2015.03.012 25937438

[B24] Marchetti O. Moreillon P. Entenza J. M. Vouillamoz J. Glauser M. P. Bille J. (2003). Fungicidal synergism of fluconazole and cyclosporine in Candida albicans is not dependent on multidrug efflux transporters encoded by the CDR1, CDR2, CaMDR1, and FLU1 genes. Antimicrob. Agents Chemother. 47, 1565–1570. 10.1128/aac.47.5.1565-1570.2003 12709323 PMC153326

[B25] Monk B. C. Goffeau A. (2008). Outwitting multidrug resistance to antifungals. Science 321, 367–369. 10.1126/science.1159746 18635793

[B26] Noble S. M. Johnson A. D. (2005). Strains and strategies for large-scale gene deletion studies of the diploid human fungal pathogen Candida albicans. Eukaryot. Cell 4, 298–309. 10.1128/EC.4.2.298-309.2005 15701792 PMC549318

[B27] Nomura S. Horiuchi T. Omura S. Hata T. (1972). The action mechanism of cerulenin. I. Effect of cerulenin on sterol and fatty acid biosynthesis in yeast. J. Biochem. 71, 783–796. 10.1093/oxfordjournals.jbchem.a129827 4561339

[B28] Pasrija R. Panwar S. L. Prasad R. (2008). Multidrug transporters CaCdr1p and CaMdr1p of Candida albicans display different lipid specificities: Both ergosterol and sphingolipids are essential for targeting of CaCdr1p to membrane rafts. Antimicrob. Agents Chemother. 52, 694–704. 10.1128/AAC.00861-07 18056285 PMC2224756

[B29] Pasrija R. Prasad T. Prasad R. (2005). Membrane raft lipid constituents affect drug susceptibilities of Candida albicans. Biochem. Soc. Trans. 33, 1219–1223. 10.1042/BST20051219 16246085

[B30] Perlin D. S. Rautemaa-Richardson R. Alastruey-Izquierdo A. (2017). The global problem of antifungal resistance: Prevalence, mechanisms, and management. Lancet Infect. Dis. 17, e383–e392. 10.1016/S1473-3099(17)30316-X 28774698

[B31] Prasad R. Balzi E. Banerjee A. Khandelwal N. K. (2019). All about CDR transporters: Past, present, and future. Yeast 36, 223–233. 10.1002/yea.3356 30192990

[B32] Prasad R. Shah A. H. Sanwal H. Kapoor K. (2012). Alanine scanning of all cysteines and construction of a functional cysteine-less Cdr1p, a multidrug ABC transporter of Candida albicans. Biochem. Biophys. Res. Commun. 417, 508–513. 10.1016/j.bbrc.2011.11.150 22166216

[B33] Prasad T. Saini P. Gaur N. A. Vishwakarma R. A. Khan L. A. Haq Q. M. (2005). Functional analysis of CaIPT1, a sphingolipid biosynthetic gene involved in multidrug resistance and morphogenesis of Candida albicans. Antimicrob. Agents Chemother. 49, 3442–3452. 10.1128/AAC.49.8.3442-3452.2005 16048959 PMC1196211

[B34] Rosenberg A. Ene I. V. Bibi M. Zakin S. Segal E. S. Ziv N. (2018). Antifungal tolerance is a subpopulation effect distinct from resistance and is associated with persistent candidemia. Nat. Commun. 9, 2470. 10.1038/s41467-018-04926-x 29941885 PMC6018213

[B35] Ryder N. S. (1992). Terbinafine: Mode of action and properties of the squalene epoxidase inhibition. Br. J. Dermatol 126 (39), 2–7. 10.1111/j.1365-2133.1992.tb00001.x 1543672

[B36] Sa N. P. Lima C. M. Lino C. I. Barbeira P. J. S. Baltazar L. M. Santos D. A. (2017). Heterocycle thiazole compounds exhibit antifungal activity through increase in the production of reactive oxygen species in the cryptococcus neoformans-cryptococcus gattii species complex. Antimicrob. Agents Chemother. 61, e02700–e02716. 10.1128/AAC.02700-16 28533240 PMC5527588

[B37] Sanglard D. Ischer F. Marchetti O. Entenza J. Bille J. (2003). Calcineurin A of Candida albicans: Involvement in antifungal tolerance, cell morphogenesis and virulence. Mol. Microbiol. 48, 959–976. 10.1046/j.1365-2958.2003.03495.x 12753189

[B38] Sanglard D. Ischer F. Monod M. Bille J. (1996). Susceptibilities of Candida albicans multidrug transporter mutants to various antifungal agents and other metabolic inhibitors. Antimicrob. Agents Chemother. 40, 2300–2305. 10.1128/AAC.40.10.2300 8891134 PMC163524

[B39] Sellam A. Askew C. Epp E. Lavoie H. Whiteway M. Nantel A. (2009). Genome-wide mapping of the coactivator Ada2p yields insight into the functional roles of SAGA/ADA complex in Candida albicans. Mol. Biol. Cell 20, 2389–2400. 10.1091/mbc.e08-11-1093 19279142 PMC2675619

[B40] Shukla S. Saini P. Smriti A. Jha S. Ambudkar S. V. Prasad R. (2003). Functional characterization of Candida albicans ABC transporter Cdr1p. Eukaryot. Cell 2, 1361–1375. 10.1128/ec.2.6.1361-1375.2003 14665469 PMC326652

[B41] Singh S. D. Robbins N. Zaas A. K. Schell W. A. Perfect J. R. Cowen L. E. (2009). Hsp90 governs echinocandin resistance in the pathogenic yeast Candida albicans via calcineurin. PLoS Pathog. 5, e1000532. 10.1371/journal.ppat.1000532 19649312 PMC2712069

[B42] Song J. Liu X. Li R. (2020). Sphingolipids: Regulators of azole drug resistance and fungal pathogenicity. Mol. Microbiol. 114, 891–905. 10.1111/mmi.14586 32767804

[B43] Suchodolski J. Muraszko J. Bernat P. Krasowska A. (2019). A crucial role for ergosterol in plasma membrane composition, localisation, and activity of Cdr1p and H(+)-ATPase in Candida albicans. Microorganisms 7, 378. 10.3390/microorganisms7100378 31546699 PMC6843828

[B44] Suchodolski J. Muraszko J. Bernat P. Krasowska A. (2021). Lactate like fluconazole reduces ergosterol content in the plasma membrane and synergistically kills Candida albicans. Int. J. Mol. Sci. 22, 5219. 10.3390/ijms22105219 34069257 PMC8156871

[B45] Teixeira V. Costa V. (2016). Unraveling the role of the Target of Rapamycin signaling in sphingolipid metabolism. Prog. Lipid Res. 61, 109–133. 10.1016/j.plipres.2015.11.001 26703187

[B46] Teo J. Q. Lee S. J. Tan A. L. Lim R. S. Cai Y. Lim T. P. (2019). Molecular mechanisms of azole resistance in Candida bloodstream isolates. BMC Infect. Dis. 19, 63. 10.1186/s12879-019-3672-5 30654757 PMC6337757

[B47] Thomas E. Roman E. Claypool S. Manzoor N. Pla J. Panwar S. L. (2013). Mitochondria influence CDR1 efflux pump activity, Hog1-mediated oxidative stress pathway, iron homeostasis, and ergosterol levels in Candida albicans. Antimicrob. Agents Chemother. 57, 5580–5599. 10.1128/AAC.00889-13 23979757 PMC3811284

[B48] Tsao S. Rahkhoodaee F. Raymond M. (2009). Relative contributions of the Candida albicans ABC transporters Cdr1p and Cdr2p to clinical azole resistance. Antimicrob. Agents Chemother. 53, 1344–1352. 10.1128/AAC.00926-08 19223631 PMC2663127

[B49] Xu D. Jiang B. Ketela T. Lemieux S. Veillette K. Martel N. (2007). Genome-wide fitness test and mechanism-of-action studies of inhibitory compounds in Candida albicans. PLoS Pathog. 3, e92. 10.1371/journal.ppat.0030092 17604452 PMC1904411

[B50] Xu Y. Lu H. Zhu S. Li W. Q. Jiang Y. Y. Berman J. (2021). Multifactorial mechanisms of tolerance to ketoconazole in Candida albicans. Microbiol. Spectr. 9, e0032121. 10.1128/Spectrum.00321-21 34160280 PMC8552639

[B51] Xu Y. Wang Y. Yan L. Liang R. M. Dai B. D. Tang R. J. (2009). Proteomic analysis reveals a synergistic mechanism of fluconazole and berberine against fluconazole-resistant Candida albicans: Endogenous ROS augmentation. J. Proteome Res. 8, 5296–5304. 10.1021/pr9005074 19754040

[B52] Yu Q. Ding X. Xu N. Cheng X. Qian K. Zhang B. (2013). *In vitro* activity of verapamil alone and in combination with fluconazole or tunicamycin against Candida albicans biofilms. Int. J. Antimicrob. Agents 41, 179–182. 10.1016/j.ijantimicag.2012.10.009 23265915

