# Supplementary figures and images for: Myriocin enhances the antifungal activity of fluconazole by blocking the membrane localization of the efflux pump Cdr1
Source: Front Pharmacol. 2022 Dec 21;13:1101553. doi: 10.3389/fphar.2022.1101553 (PMC9815617; doi:10.3389/fphar.2022.1101553)

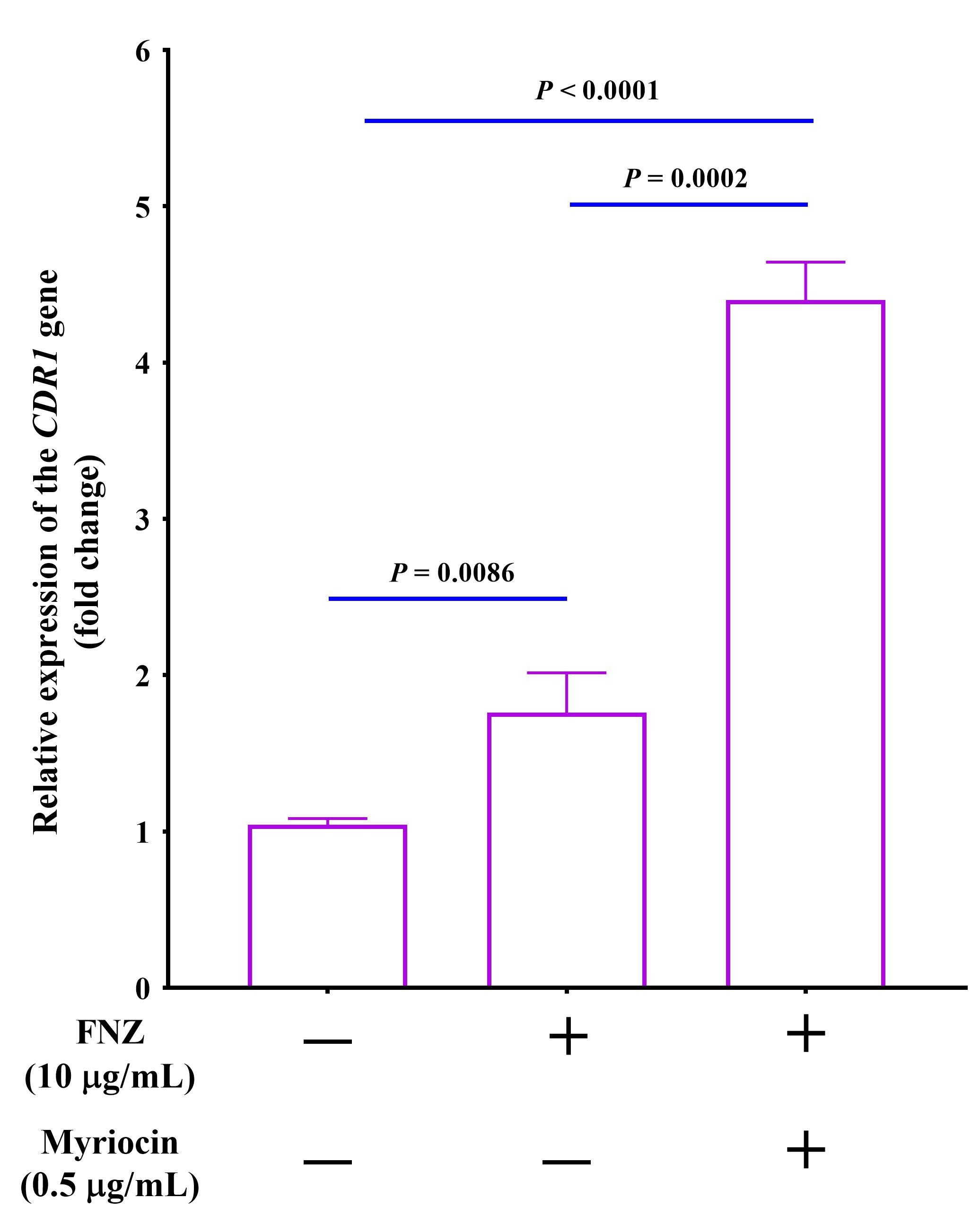

Supplement: Supplementary file 2 [file Image3.JPEG]

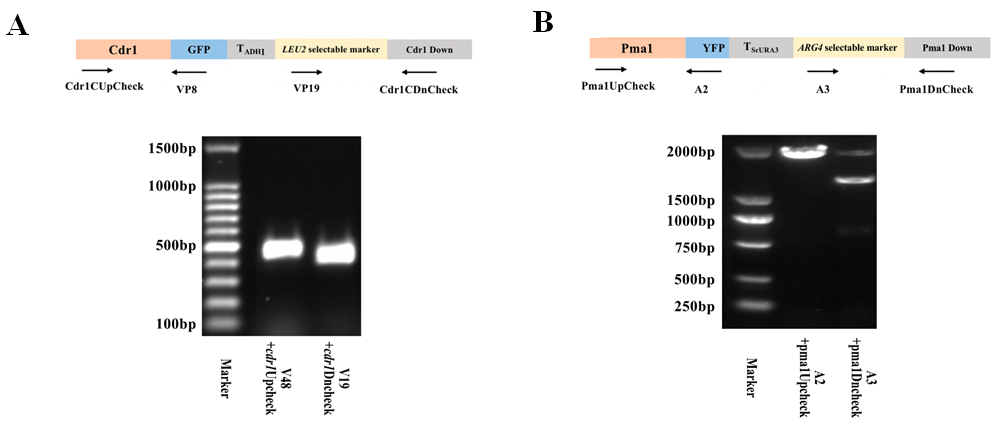

Supplement: Supplementary file 3 [file Image4.TIF]

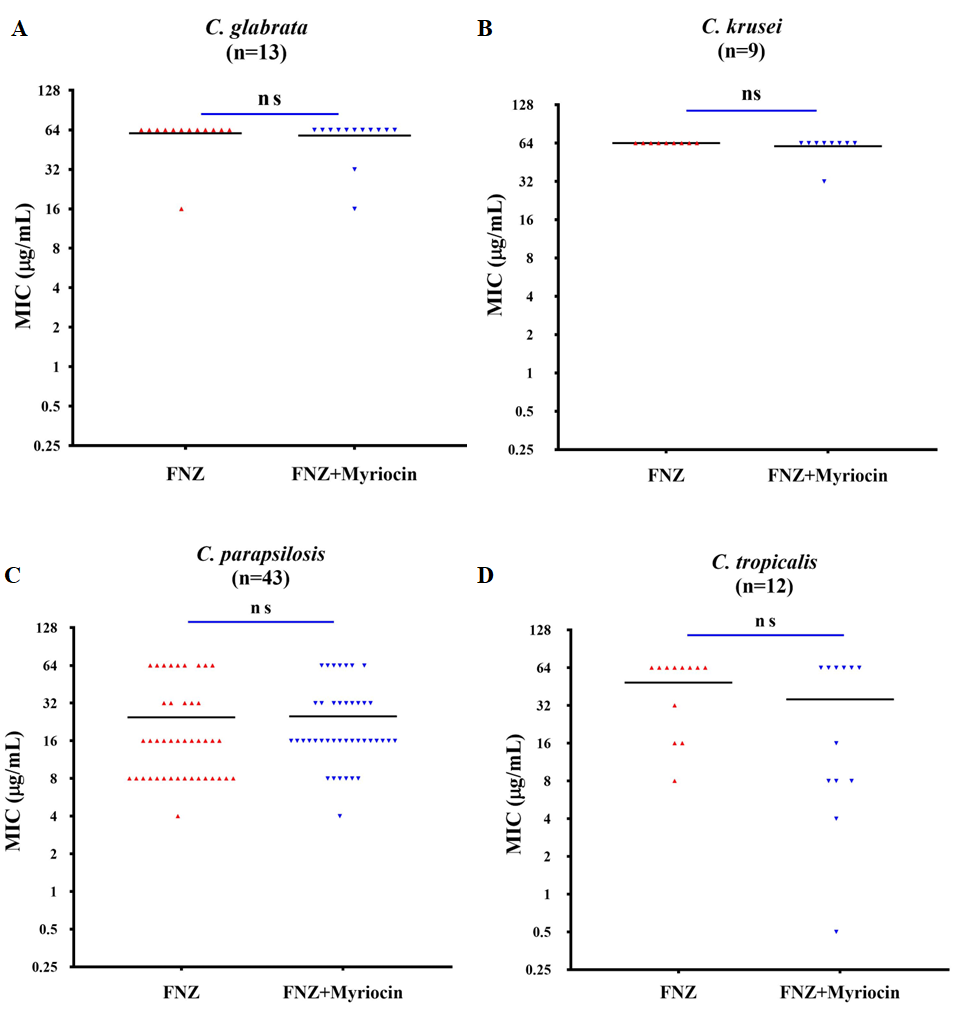

Supplement: Supplementary file 4 [file Image2.TIF]

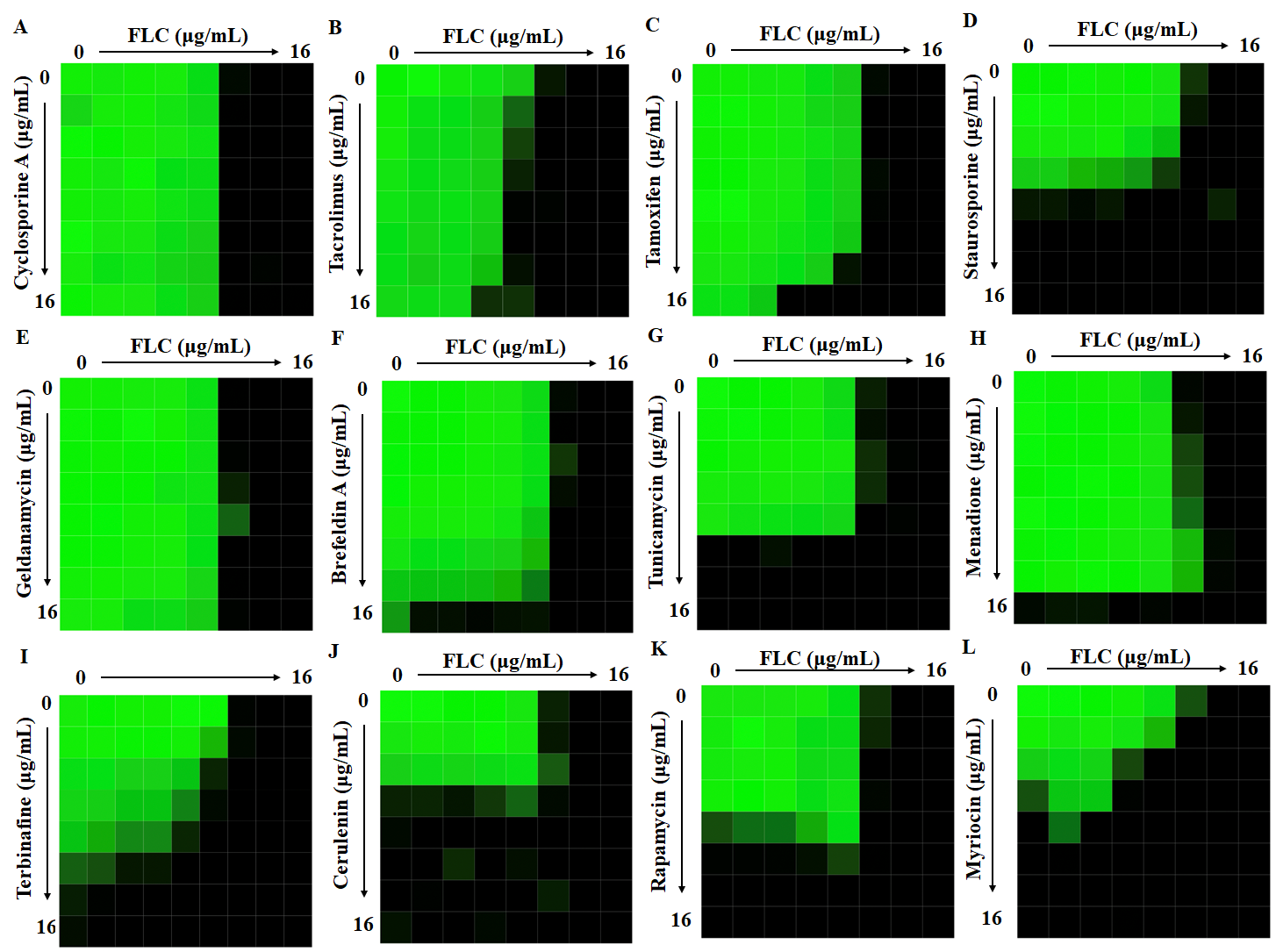

Supplement: Supplementary file 5 [file Image1.TIF]

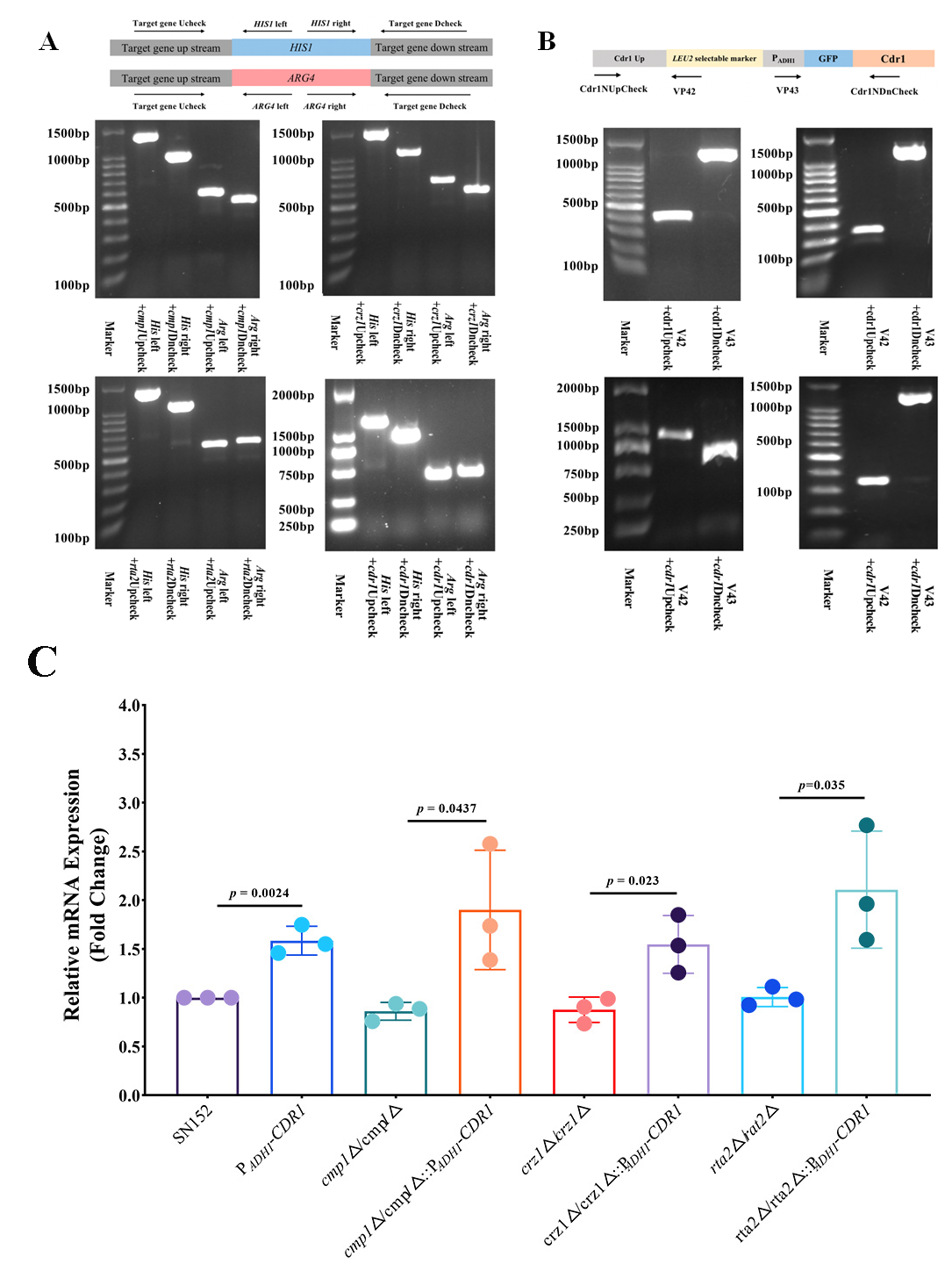

Supplement: Supplementary file 7 [file Image5.TIF]
